# A pan-cancer analysis of the prognostic value of long non-coding RNA LINC00662 in human cancers

**DOI:** 10.3389/fgene.2022.1063119

**Published:** 2022-12-08

**Authors:** Guangming Zhang, Bin Wu, Liangyin Fu, Bin Liu, Xiaoyong Han, Jie Wang, Yipeng Zhang, Miao Yu, Haizhong Ma, Shixun Ma, Hui Cai

**Affiliations:** ^1^ The First Clinical Medical College of Gansu University of Chinese Medicine (Gansu Provincial Hospital), Lanzhou, China; ^2^ Department of General Surgery, Clinical Medical Center, Gansu Provincial Hospital, Lanzhou, China; ^3^ Key Laboratory of Molecular Diagnostics and Precision Medicine for Surgical Oncology in Gansu Province, Lanzhou, China; ^4^ Gansu Provincial Hospital, Lanzhou, China; ^5^ The First Clinical Medical College of Lanzhou University, Lanzhou, China; ^6^ Ning Xia Medical University, Yinchuan, China

**Keywords:** LINC00662, meta-analysis, cancers, prognosis, bioinformatics

## Abstract

**Background:** Numerous studies have revealed that the long non-coding RNA LINC00662 is irregularly expressed in various cancers, as well as is correlated with cancer development and progression. Nevertheless, the clinical value of LINC00662 remains controversial. Hence, we explored the correlation of LINC00662 with cancer prognosis through meta-analysis and bioinformatics analysis.

**Methods:** From the beginning through 12 March 2022, we searched for correlational studies on Web of Science, Embase, PubMed and The Cochrane Library. We used pooled hazard ratios (HRs) and odds ratios (ORs) with 95% confidence intervals (CIs) to determine the significance of studies on survival outcomes and clinicopathological aspects in human cancers. Additionally, the Gene Expression Profiling Interactive Analysis (GEPIA) database was employed to confirm our findings.

**Results:** Our meta-analysis of 14 studies comprising a total of 960 cancer patients revealed that LINC00662 overexpression was correlated with poor overall survival (HR = 1.91, 95% CI 1.49–2.45, *p* < 0.001) in cancer patients and relapse-free survival (HR = 2.12, 95% CI 1.19–3.76, *p* = 0.010) in hepatocellular carcinoma patients. The correlation between LINC00662 and OS was further supported by the results of subgroup analyses according to cancer type, follow-up time, HR availability, and NOS score. In addition, LINC00662 overexpression predicted advanced tumor stage (OR = 4.23, 95% CI 2.50–7.17, *p* < 0.001), larger tumor size (OR = 1.49, 95% CI 1.11–1.99, *p* = 0.008), earlier lymph node metastasis (OR = 2.40, 95% CI 1.25–4.59, *p* = 0.008), and earlier distant metastasis (OR = 4.78, 95% CI 2.57–8.88, *p* < 0.001). However, there were no statistically significant differences in age (OR = 1.16, 95% CI 0.90–1.51, *p* = 0.246), gender (OR = 1.10, 95% CI 0.79–1.53, *p* = 0.578), or differentiation grade (OR = 1.53, 95% CI 0.71–3.33, *p* = 0.280).

**Conclusion:** LINC00662 expression upregulation is associated with poor prognosis and advanced clinicopathological features in patients with multiple tumors. LINC00662 may serve as a biomarker for the diagnosis and treatment of patients with tumors.

## Introduction

Cancer is one of the leading causes of death globally, and the worldwide cancer burden is increasing (Sung et al., 2021). Despite considerable advancement in modern cancer treatments, such as surgical therapy and radiotherapy, many patients still progress to the advanced cancer stage, which results in a lousy prognosis (Chen et al., 2021). The lack of clear markers for tumor diagnosis is among the fundamental causes for this dismal prognosis (Avila et al., 2006). Consequently, finding new tumor indicators for diagnosis, prognosis, and treatment is crucial.

Long non-coding RNA (lncRNA) is over 200-nucleotide long, of which 55 nucleotides lack a specific open reading frame, rendering the encoded protein inactive (Xue et al., 2017). Although lncRNAs were initially considered transcriptional disruptions, they are recently implicated in various diseases (Renganathan and Felley-Bosco, 2017). Recently, an increasing amount of lncRNAs was found to be inappropriately expressed in cancer and was implicated in diverse pathophysiological processes, such as controlling gene expression through epigenetic, transcription, and post-transcription modification (Lin et al., 2021). Therefore, lncRNAs are potentially both tumorigenic and antitumorigenic (Huarte and Rinn, 2010). Because of their unique expression and functionality in multiple cancer types, lncRNAs act as promising cancer diagnostic, prognostic, and therapeutic biomarkers (Wang et al., 2021).

LINC00662, the long intergenic non-protein coding RNA 662, is located on chromosome 19q11 and is 2085-bp long (Zhong et al., 2021). Aberrant LINC00662 expression was first reported in lung tissues and cells (Gong et al., 2018). Many recent studies have found that LINC00662 is overexpressed in numerous tumors, and this overexpression implies poor prognosis, such as prostate cancer (PCa) (Li et al., 2019), colorectal cancer (CRC) (Wang et al., 2019; Cheng B. et al., 2020), bladder cancer (Ma et al., 2021), osteosarcoma (OSA) (Yu et al., 2021), hepatocellular carcinoma (HCC) (Guo et al., 2020; Tian et al., 2020), ovarian cancer (OC) (Wu et al., 2021), cervical cancer (CC) (Wei et al., 2020), glioma (Geng et al., 2020; Wu et al., 2020), and gastric cancer (GC) (Liu et al., 2018). Furthermore, elevated LINC00662 expression is usually linked to advanced clinicopathological features. For instance, in breast cancer (BC) (Cheng L. et al., 2020), high LINC00662 expression is related to the advanced tumor stage and positive lymph node metastasis (LNM). Moreover, LINC00662 could be engaged in tumor proliferation, apoptosis, invasion and migration (Cheng B. et al., 2020; Liu and Meng, 2020; Xu et al., 2021). Some studies have confirmed the critical link between LINC00662 and tumor-related signaling pathways (Liu Y. L. et al., 2021; Yu et al., 2021). Taken together, LINC00662 could be a prospective prognostic marker and therapeutic target for most human malignancies. However, most studies are restricted by the small sample size or scattered results, and the mechanism of action has not yet been entirely clarified. Therefore, we performed quantitative meta-analysis and bioinformatics analysis to estimate the combined role of LINC00662 in human cancers.

## Materials and methods

### Search strategy and literature collection

The registry was established with PROSPERO prior to the start of the research (registration number: CRD42021286741). A structured literature search of Web of Science, Embase, PubMed and The Cochrane Library was performed from their inception through 12 March 2022. “Long intergenic non-protein coding RNA 662,” “lncRNA LINC00662,” “LINC00662,” “neoplasm,” “cancer,” “malignancy,” “neoplasia,” “melanoma,” “tumor,” “sarcoma,” “carcinoma” or “adenoma” were the search terms. Relevant study citation lists were also thoroughly reviewed. To ensure accuracy and consistency, two investigators individually assessed the database search methodologies and debated the results.

### Inclusion and exclusion criteria

The inclusion criteria were as follows: 1) discussing LINC00662 expression in cancer tissues; 2) in which patients were classified into two groups by the LINC00662 expression levels; 3) reporting the connection between LINC00662 expression and cancer prognosis or clinicopathological characteristics; and 4) in which hazard ratios (HRs) and 95% confidence intervals (CIs) for survival data were presented or could be obtained from survival curves.

The exclusion criteria were as follows: 1) conference reports, *in vitro* or *in vivo* experiments, and reviews; 2) duplicate publications; and 3) articles whose data could not be extracted and articles from non-English publications.

### Data extraction and quality assessment

Two researchers separately explored and chose articles based on the aforementioned criteria, extracting the following data: first author’s name, publication year, country of study, types of cancer, number of patients, detection methods, cut-off criteria, clinical parameters, overall survival (OS), relapse-free survival (RFS), disease-free survival (DFS), and progression-free survival (PFS). Studies were used immediately if they contained precise survival data. When publications supplied only Kaplan Meier (KM) curves without precise survival data, we used Engauge Digitizer V.4.1 software to obtain data from the KM curves and computed HRs and 95% CIs. Literature quality was evaluated using the Newcastle-Ottawa Scale (NOS). The study was graded on a nine-item scale, with one point assigned for each item completed. Aggregate scores varied from 0 to 9. The NOS score of 7 and above denoted high-quality research findings.

### Validation by using public datasets

Gene Expression Profiling Interactive Analysis (GEPIA), an online web-based tool based on TCGA and GTEx data, is designed to verify the LINC00662 expression levels in pan-cancer. LINC00662-related survival analysis was conducted using the KM method and log-rank test.

### Prediction of LINC00662 functions and pathways

The MEM-Multi Experiment Matrix database, which is based on the Affymetrix Gene Chip Human Genome U133 Plus 2.0 Array technology, was used to identify the relevant genes of LINC00662. Then, gene ontology (GO) analysis and Kyoto Encyclopedia of Genes and Genomes (KEGG) pathway analysis were performed using R software. Finally, Cytoscape software was utilized for constructing the signaling pathway network.

### Statistical analysis

Stata 12.0 was used to estimate all statistical data. To investigate the relationship between LINC00662 expression and survival outcome, HRs with 95%CIs were determined. To investigate the link between LINC00662 expression and clinicopathological features, researchers estimated pooled odds ratios (ORs) with 95% CIs. If heterogeneity was moderate (*P*_*Q*_ > 0.1, *I* ^*2*^ < 50%), a fixed-effects model was applied. Otherwise, a random-effects model was applied. Significant heterogeneity was addressed using methods such as subgroup analysis or sensitivity analysis. All data were presented in the form of Forest plots. Egger’s test and Begg’s funnel plot were used to determine possible publication bias, while sensibility analysis was employed to determine the causes of heterogeneity and to verify the stability of the results. *p* < 0.05 was considered statistically significant.

## Results

### Characteristics of studies

**FIGURE 1:**
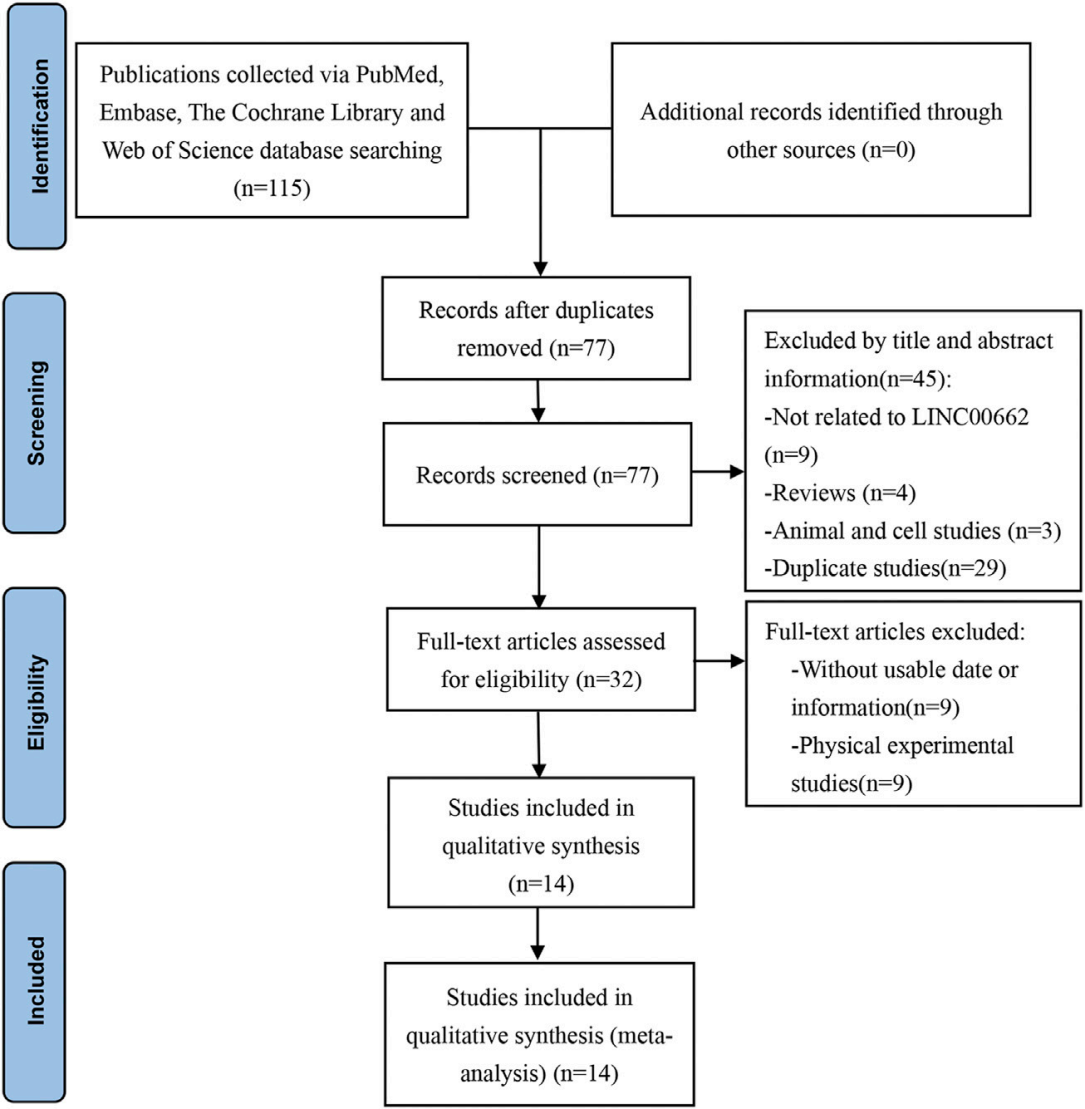
Flow diagram of this meta-analysis.

In total, 115 studies were initially obtained according to the search strategy (Web of Science = 35, Embase = 44, PubMed = 36, Cochrane = 0). After removing 38 duplicate studies, 45 articles were removed as extraneous papers by scanning the titles and abstracts. The remaining 32 studies were then carefully reviewed by reading the full article. Of them, 9 were eliminated due to the lack of eligible data, 9 were eliminated since they were physical experimental studies, and 14 studies (Gong et al., 2018; Li et al., 2019; Wang et al., 2019; Cheng B. et al., 2020; Cheng L. et al., 2020; Guo et al., 2020; Liu and Meng, 2020; Tian et al., 2020; Wei et al., 2020; Zhang et al., 2020; Ma et al., 2021; Wu et al., 2021; Yu et al., 2021; Wang et al., 2022) were finally included for meta-analysis. The study screening procedure is presented in Figure 1.

The 14 enrolled articles contained a total of 960 patients, with sample sizes between 39 and 105. All of these articles were conducted in China and published between 2018 and 2022. The above articles included ten tumors, including three cases of OSA (Liu and Meng, 2020; Yu et al., 2021; Wang et al., 2022), two cases of HCC (Guo et al., 2020; Tian et al., 2020), two cases of CRC (Wang et al., 2019; Cheng B. et al., 2020), one case of bladder cancer (Ma et al., 2021), one case of lung cancer (Gong et al., 2018), one case of CC (Wei et al., 2020), one case of ESCC (Zhang et al., 2020), one case of BC (Cheng L. et al., 2020), one case of PCa (Li et al., 2019), and one case of OC (Wu et al., 2021). Nine of these articles (Gong et al., 2018; Li et al., 2019; Wang et al., 2019; Cheng B. et al., 2020; Guo et al., 2020; Tian et al., 2020; Ma et al., 2021; Wu et al., 2021; Yu et al., 2021) reported OS, two (Guo et al., 2020; Tian et al., 2020) reported RFS, and all studies reported clinical parameters. Three (Li et al., 2019; Guo et al., 2020; Wu et al., 2021) OS data and one (Guo et al., 2020) RFS data were obtained directly from the original text, and six (Gong et al., 2018; Wang et al., 2019; Cheng B. et al., 2020; Tian et al., 2020; Ma et al., 2021; Yu et al., 2021) OS data and one (Tian et al., 2020) RFS data were extracted by KM curves. All studies employed real-time quantitative polymerase chain reaction (RT-qPCR) to detect the expression levels of LINC00662. The characteristics of the included articles are summarized in Table 1.

**TABLE 1:** Characteristics of studies in this meta-analysis.

Study	Year	Country	Cancer type	Sample type	Total Size(n)	Detection method	Cutoff	Outcome	Multivariate analysis	HR statistic	Follow-up times (month)	NOS score
Cheng B	2020	China	colon cancer	Tissue	72	RT-qPCR	mean	CP/OS	No	SC	36	7
Cheng L	2020	China	BC	Tissue	47	RT-qPCR	median	CP	No	NA	NA	6
Gong	2018	China	LC	Tissue	70	RT-qPCR	NA	CP/OS	No	SC	48	6
Guo	2020	China	HCC	Tissue	70	RT-qPCR	NA	CP/OS/RFS	Yes	Rep	60	7
Li	2019	China	PCa	Tissue	105	RT-qPCR	median	CP/OS	Yes	Rep	60	8
Liu	2020	China	OSA	Tissue	57	RT-qPCR	median	CP	No	NA	NA	6
Ma	2021	China	bladder cancer	Tissue	104	RT-qPCR	NA	CP/OS	No	SC	36	6
Tian	2020	China	HCC	Tissue	86	RT-qPCR	median	CP/OS/RFS	No	SC	60	7
Wang	2022	China	OSA	Tissue	56	RT-qPCR	NA	CP	No	NA	NA	6
Wang	2019	China	CRC	Tissue	56	RT-qPCR	median	CP/OS	No	SC	90	7
Wei	2020	China	CC	Tissue	39	RT-qPCR	NA	CP	No	NA	NA	6
Wu	2021	China	OC	Tissue	75	RT-qPCR	median	CP/OS	Yes	Rep	80	8
Yu	2021	China	OSA	Tissue	51	RT-qPCR	median	CP/OS	Yes	SC	60	8
Zhang	2020	China	ESCC	Tissue	72	RT-qPCR	NA	CP	No	NA	NA	6

HR, hazard ratio; BC, breast cancer; LC, lung cancer; HCC, hepatocellular carcinoma; PCa, prostate cancer; OSA, osteosarcoma; CRC, colorectal cancer; CC, cervical cancer; OC, ovarian cancer; ESCC, esophageal squamous cell carcinoma; NA, not available; CP, clinicopathological parameters; OS, overall survival; RFS, relapse free survival; SC, survival curve; Rep, report in text; RT-qPCR, real-time quantitative polymerase chain reaction; NOS, Newcastle–Ottawa Scale.

### Association between LINC00662 expression and survival

**FIGURE 2:**
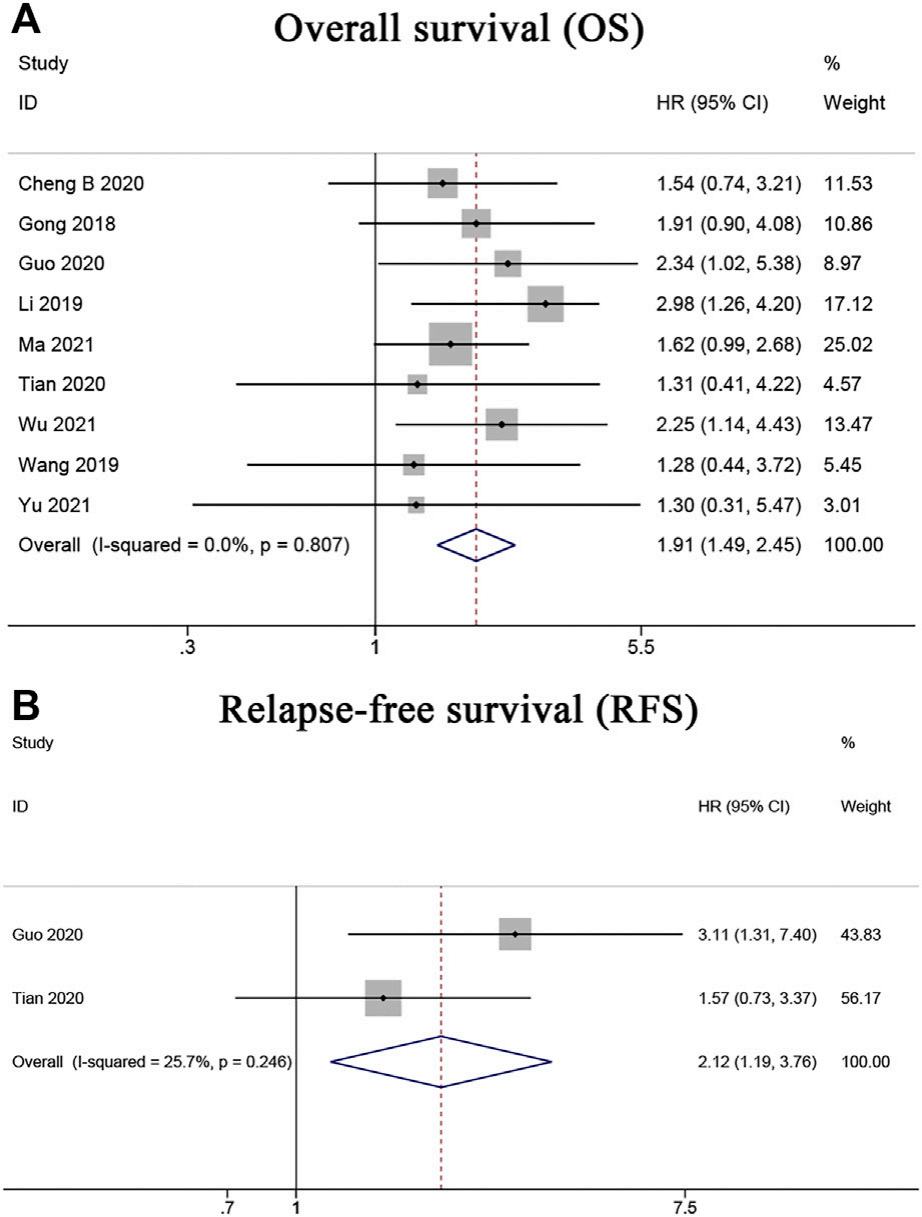
Forest plots for association of LINC00662 expression with survival. **(A)** Overall survival. **(B)** Relapse-free survival.

Nine articles (Gong et al., 2018; Li et al., 2019; Wang et al., 2019; Cheng B. et al., 2020; Guo et al., 2020; Tian et al., 2020; Ma et al., 2021; Wu et al., 2021; Yu et al., 2021) including 689 cancer patients reported a relationship between abnormal LINC00662 expression levels and OS. A fixed-effects model was used because no obvious heterogeneity existed (*I* ^*2*^ = 0%, *P*_*Q*_ = 0.807). As shown in Figure 2A, the HR of the pooled OS was 1.91, indicating that LINC00662 overexpression was notably interrelated with poor OS. Furthermore, two articles (Guo et al., 2020; Tian et al., 2020), with a total of 156 patients with HCC, provided suitable data for RFS. A fixed-effects model was used as no obvious heterogeneity existed (*I* ^*2*^ = 25.7%, *P*_*Q*_ = 0.246). LINC00662 overexpression was markedly interrelated with shorter RFS in patients with HCC (HR = 2.12, 95% CI 1.19–3.76, *p* = 0.010) (Figure 2B).

**FIGURE 3:**
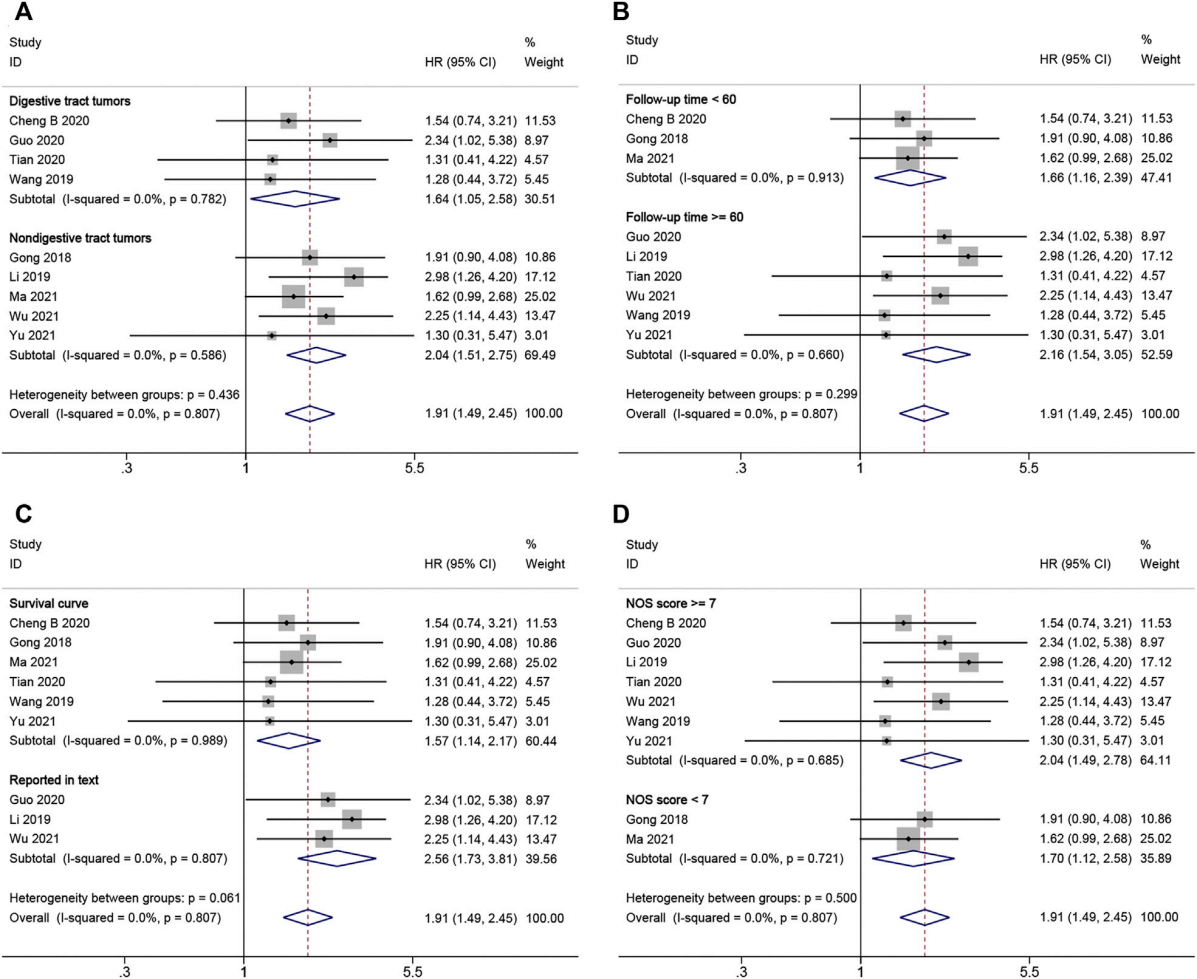
Forest plots for subgroup analysis of LINC00662 expression with overall survival. **(A)** Subgroup analysis stratified by cancer type. **(B)** Subgroup analysis stratified by follow-up time. **(C)** Subgroup analysis stratified by HR availability. **(D)** Subgroup analysis stratified by NOS score.

In addition, we conducted a subgroup meta-analysis based on tumor type (digestive tract tumors and non-digestive tract tumors), follow-up time (≥60 months or <60 months), HR availability (reported in text and survival curve), and NOS score (NOS scores ≥7 or <7) to investigate further the correlation between LINC00662 expression levels and OS. According to all subgroup analyses, revealed that LINC00662 overexpression was notably interrelated with poor OS (Figure 3; Table 2). The aforementioned results imply that LINC00662 expression levels is probably a prognostic factor for OS in patients with tumors.

**TABLE 2:** Subgroup meta-analysis of pooled HRs for OS.

Stratified analysis	Studies (n)	Number of patients	Pooled HR (95% CI)	*p*-value	Heterogeneity		
					*I* ^*2*^ (%)	*p*-value	Model
Cancer type							
Digestive tract tumors	4	284	1.64 (1.05–2.58)	0.031	0.0	0.782	Fixed effects
Non-digestive tract tumors	5	405	2.04 (1.51–2.75)	<0.001	0.0	0.586	Fixed effects
Follow-up time							
≥60	6	443	2.16 (1.54–3.05)	<0.001	0.0	0.660	Fixed effects
<60	3	246	1.66 (1.16–2.39)	0.006	0.0	0.913	Fixed effects
HR availability							
Survival curve	6	439	1.57 (1.14–2.17)	0.005	0.0	0.989	Fixed effects
Reported in text	3	250	2.56 (1.73–3.81)	<0.001	0.0	0.807	Fixed effects
NOS score							
≥7	7	515	2.04 (1.49–2.78)	<0.001	0.0	0.685	Fixed effects
<7	2	174	1.70 (1.12–2.58)	0.012	0.0	0.721	Fixed effects

HR, hazard ratio; CI, confidence interval; NOS, Newcastle–Ottawa Scale.

### Association between LINC00662 and clinicopathological parameters

**FIGURE 4:**
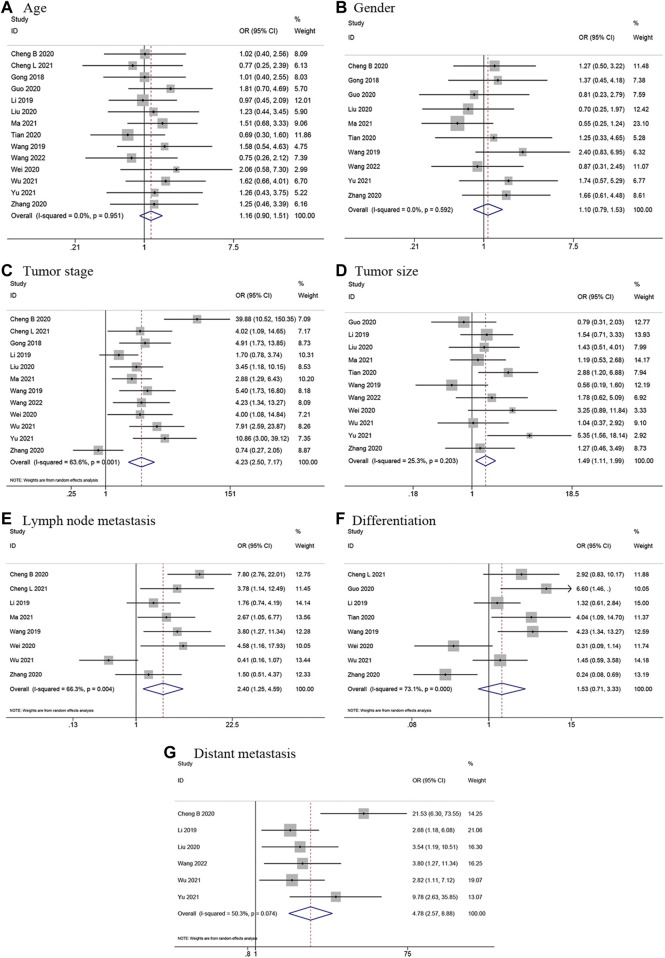
Forest plots for association of LINC00662 expression with clinicopathological features. **(A)** Age. **(B)** Gender. **(C)** Tumor stage. **(D)** Tumor size. **(E)** Lymph node metastasis. **(F)** Differentiation. **(G)** Distant metastasis.

In total, 14 articles (Gong et al., 2018; Li et al., 2019; Wang et al., 2019; Cheng B. et al., 2020; Cheng L. et al., 2020; Guo et al., 2020; Liu and Meng, 2020; Tian et al., 2020; Wei et al., 2020; Zhang et al., 2020; Ma et al., 2021; Wu et al., 2021; Yu et al., 2021; Wang et al., 2022) presented usable data regarding the link between LINC00662 expression levels and clinicopathological parameters, which included age, sex, tumor stage, tumor size, LNM, differentiation grade, and distant metastasis (DM). The analysis was conducted using ORs and their 95% CIs. According to the results, LINC00662 overexpression was notably interrelated with advanced tumor stage (OR = 4.23, 95% CI 2.50–7.17, *p* < 0.001), larger tumor size (OR = 1.49, 95% CI 1.11–1.99, *p* = 0.008), earlier LNM (OR = 2.40, 95% CI 1.25–4.59, *p* = 0.008), and earlier DM (OR = 4.78, 95% CI 2.57–8.88, *p* < 0.001). However, the differences in terms of age (OR = 1.16, 95% CI 0.90–1.51, *p* = 0.246), gender (OR = 1.10, 95% CI 0.79–1.53, *p* = 0.578), and differentiation grade (OR = 1.53, 95% CI 0.71–3.33, *p* = 0.280) were not statistically significant. The results were presented in Figure 4 and Table 3.

**TABLE 3:** Association of LINC00662 expression with clinicopathological features.

Clinicopathological parameters	Studies	Patients (n)	OR (95% CI)	*p*-value	Heterogeneity (*I* ^*2*^, *P*)	Model
Age (old vs. young)	14	960	1.16 (0.90–1.51)	0.246	0.0%, 0.951	Fixed
Gender (male vs. female)	10	694	1.10 (0.79–1.53)	0.578	0.0%, 0.592	Fixed
Tumor stage (III + IV vs. I + II)	12	804	4.23 (2.50–7.17)	<0.001	63.6%, 0.001	Random
Tumor size (big vs. small)	11	771	1.49 (1.11–1.99)	0.008	25.3%, 0.203	Fixed
LNM (positive vs. negative)	8	570	2.40 (1.25–4.59)	0.008	66.3%, 0.004	Random
Differentiation (poor vs. well/moderate)	8	550	1.53 (0.71–3.33)	0.280	73.1%, 0.000	Random
Distant metastasis (yes vs. no)	6	416	4.78 (2.57–8.88)	<0.001	50.3%, 0.074	Random

OR, odds ratio; CI, confidence interval; LNM, lymph node metastasis.

### Sensitivity analysis

**FIGURE 5:**
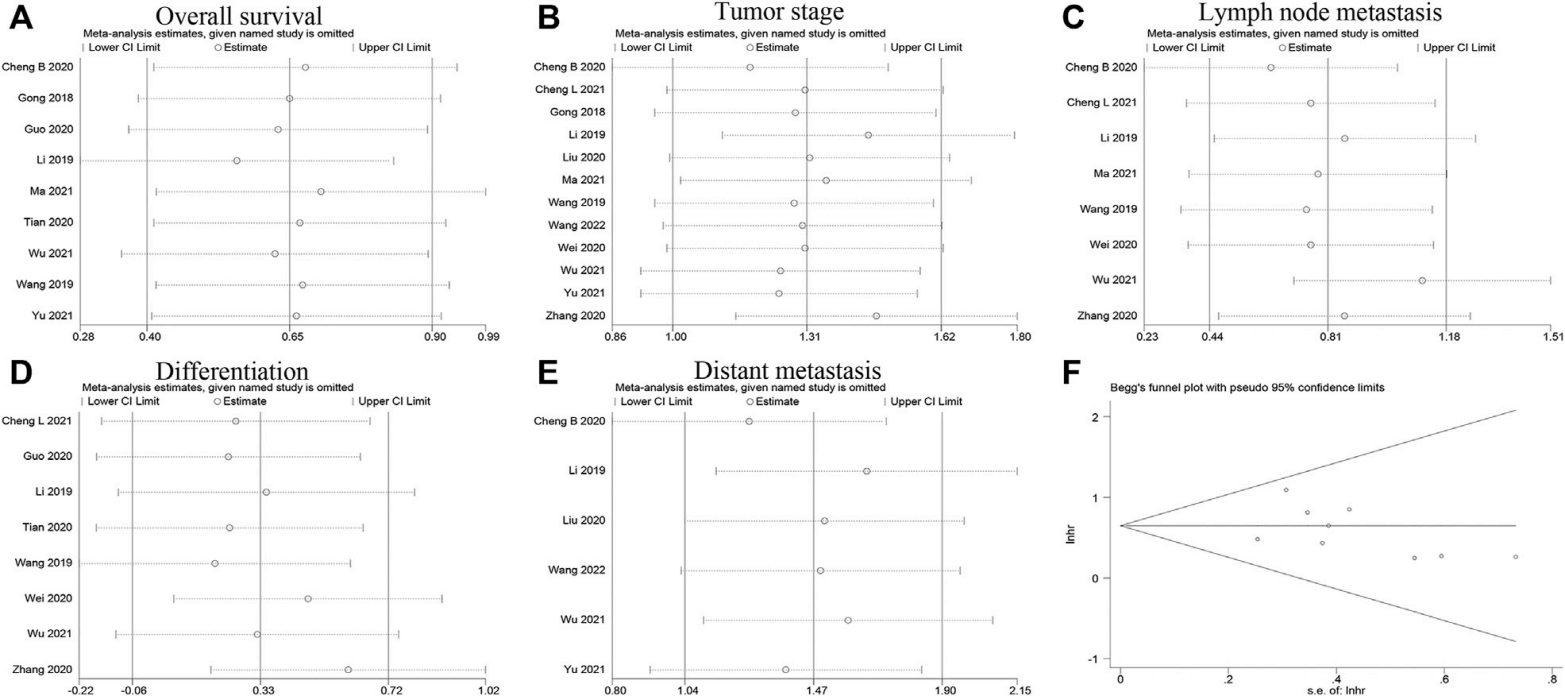
Sensitivity analysis and Begg’s funnel plot of LINC00662. **(A)** Sensitivity analysis of pooled HR for overall survival. **(B)** Sensitivity analysis of pooled HR for tumor stage. **(C)** Sensitivity analysis of pooled HR for lymph node metastasis. **(D)** Sensitivity analysis of pooled HR for differentiation. **(E)** Sensitivity analysis of pooled HR for distant metastasis. **(F)** Begg’s funnel plot of LINC00662 for overall survival.

To assess the reliability of LINC00662 expression and OS relevance findings, we conducted sensitivity analysis. No single study was found to alter the results (Figure 5A), and therefore, high LINC00662 expression was reliably associated with poor OS. Meanwhile, we performed sensitivity analysis by using the results of clinicopathological parameters. The heterogeneity of the relevance of LINC00662 overexpression to the tumor stage (Figure 5B) was completely eliminated after excluding the study of Cheng et al. (Cheng B. et al., 2020) and Zhang et al. (Zhang et al., 2020) (*P*_*Q*_ = 0.427, *I* ^*2*^ = 1.2%), without affecting the results (OR = 3.84, 95% CI 2.76–5.35, *p* < 0.001). In a sensitivity analysis of LNM (Figure 5C), excluding the study of Wu et al. (Wu et al., 2021) significantly reduced heterogeneity (*P*_*Q*_ = 0.337, *I* ^*2*^ = 12.2%), but did not affect the final results (OR = 3.02, 95% CI 2.02–4.50, *p* < 0.001). For the sensitivity analysis on differentiation grade (Figure 5D), heterogeneity was significantly reduced with the elimination of the studies of Wei et al. (Wei et al., 2020) and Zhang et al. (Zhang et al., 2020) (*P*_*Q*_ = 0.265, *I* ^*2*^ = 22.5%), and a significant correlation was observed between high LINC00662 expression and the degree of tumor differentiation (OR = 2.29, 95% CI 1.49–3.52, *p* < 0.001). Furthermore, sensitivity analysis of the relevance of LINC00662 overexpression to DM (Figure 5E) displayed complete elimination of heterogeneity after the exclusion of the study of Cheng et al. (Cheng B. et al., 2020) (*P*_*Q*_ = 0.578, *I* ^*2*^ = 0.0%), but the results were not affected (OR = 3.54, 95% CI 2.25–5.56, *p* < 0.001).

### Analysis of publication bias

Additionally, we used Begg’s funnel plot and Egger’s regression test to analyze the publication bias of the included studies on the correlation between LINC00662 expression and OS. Neither Begg’s funnel plot (Figure 5F) nor Egger’s regression test (Pr > |t| = 0.307) found significant publication bias, suggesting that our results are plausible.

### Validation of LINC00662 results in public databases

**FIGURE 6:**
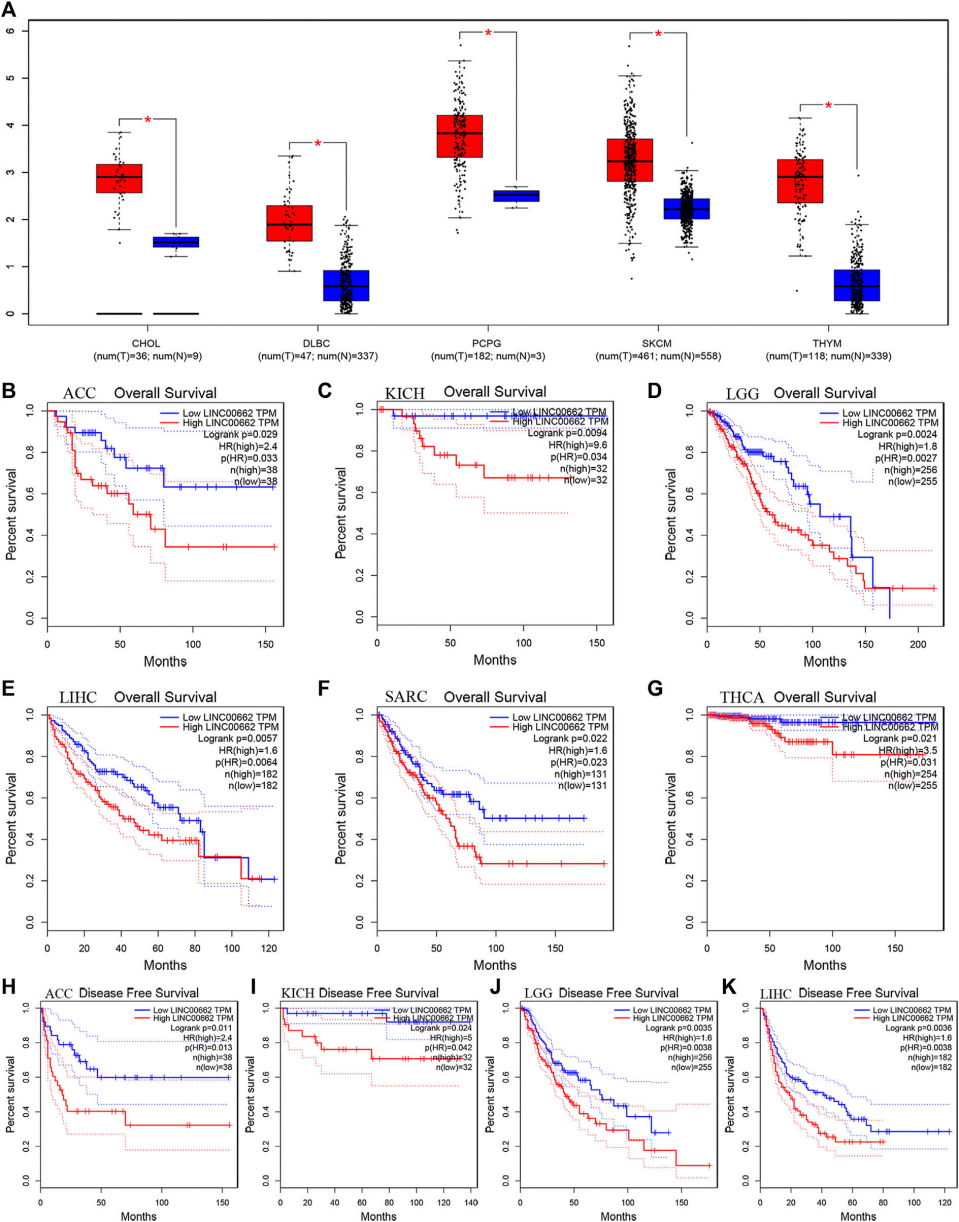
Validation of the role of LINC00662 in human cancers based on the GEPIA cohort. **(A)** The expression of LINC00662 in human cancers (red box) and normal tissues (blue box) based on the TCGA and GTEx databases (|Log_2_FC|>1 and *p* < 0.05). **(B)** OS plots of LINC00662 in ACC. **(C)** OS plots of LINC00662 in KICH. **(D)** OS plots of LINC00662 in LGG. **(E)** OS plots of LINC00662 in LIHC. **(F)** OS plots of LINC00662 in SARC. **(G)** OS plots of LINC00662 in THCA. **(H)** DFS plots of LINC00662 in ACC. **(I)** DFS plots of LINC00662 in KICH. **(J)** DFS plots of LINC00662 in LGG. **(K)** DFS plots of LINC00662 in LIHC. CHOL (cholangiocarcinoma), DLBC (lymphoid neoplasm diffuse large B-cell lymphoma), PCPG (pheochromocytoma and paraganglioma), SKCM (skin cutaneous melanoma), THYM (thymoma), OS (overall survival), ACC (adrenocortical carcinoma), KICH (kidney chromophobe), LGG (brain lower grade glioma), LIHC (liver hepatocellular carcinoma), SARC (sarcoma), THCA (thyroid carcinoma), DFS (disease-free survival).

To further confirm the reliability of our results, we assessed the relationship between LINC00662 expression in pan-cancer and prognosis of cancer patients by using the GEPIA database. Regarding aberrant LINC00662 expression, LINC00662 was highly expressed in cholangiocarcinoma (CHOL), lymphoid neoplasm diffuse large B-cell lymphoma (DLBC), pheochromocytoma and paraganglioma (PCPG), skin cutaneous melanoma (SKCM), and thymoma (THYM) (|log_2_FC| Cutoff: 1, *p*-value: 0.01, Figure 6A). Regarding the relationship between LINC00662 expression and prognosis, LINC00662 overexpression was notably interrelated with worse OS in adrenocortical carcinoma (ACC; Figure 6B), kidney chromophobe (KICH; Figure 6C), brain lower grade glioma (LGG; Figure 6D), liver hepatocellular carcinoma (LIHC; Figure 6E), sarcoma (SARC; Figure 6F), and thyroid carcinoma (THCA; Figure 6G), and shorter DFS in ACC (Figure 6H), KICH (Figure 6I), LGG (Figure J), and LIHC (Figure 6K).

### LINC00662 function and pathway prediction

**FIGURE 7:**
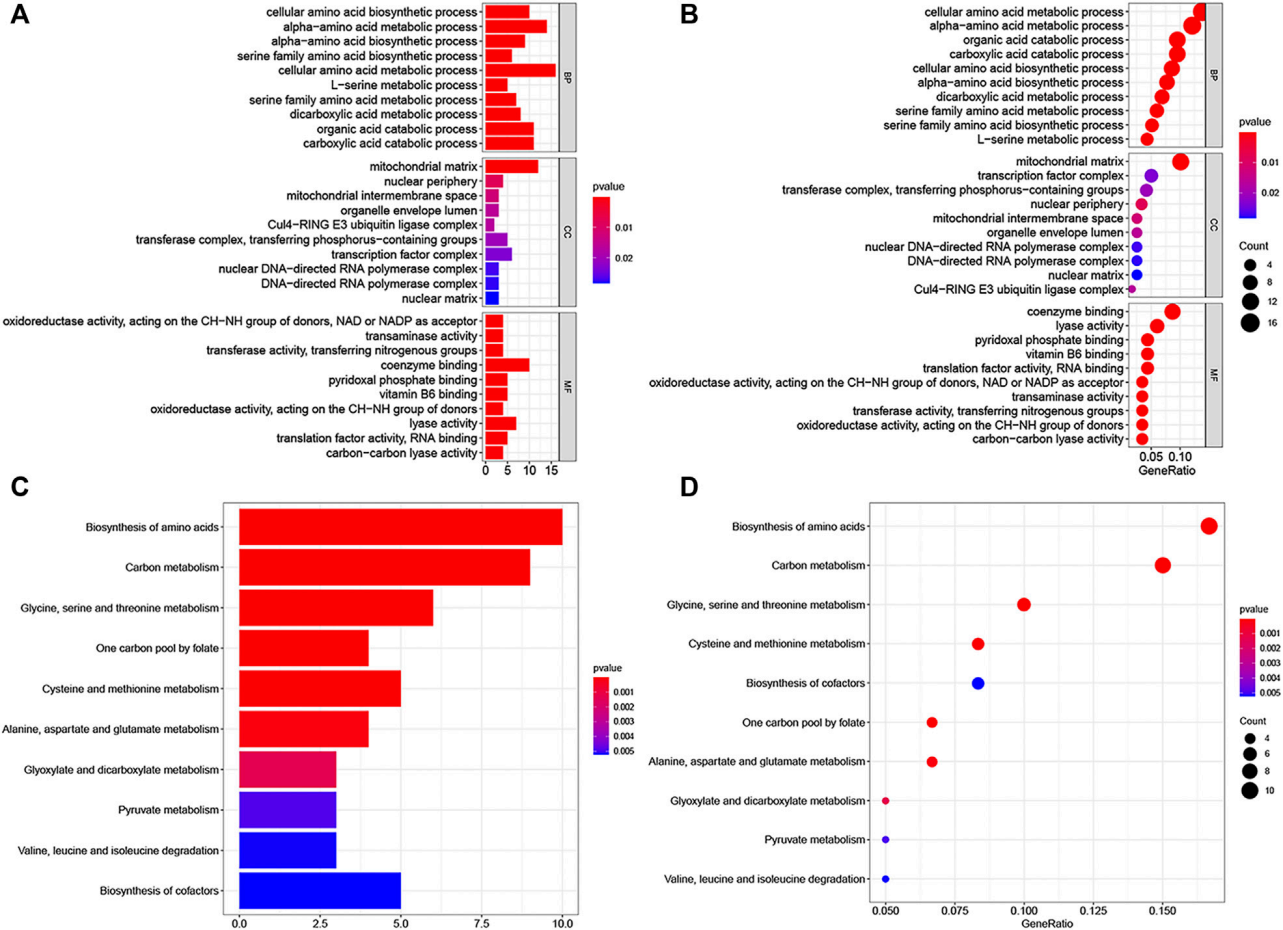
GO terms and the KEGG pathway. **(A)** Histogram presentation of GO enrichment of LINC00662-related genes in biological processes (BP), cellular components (CC) and molecular functions (MF) of top 10 terms; **(B)** Bubble chart of GO enrichment of LINC00662-related genes in the aspects of BP, CC, MF of top 10 terms; **(C)** Histogram presentation of top 10 significant pathways related to the LINC00662-related genes by the KEGG pathway analysis; **(D)** Bubble chart of top 10 significant pathways.

**FIGURE 8:**
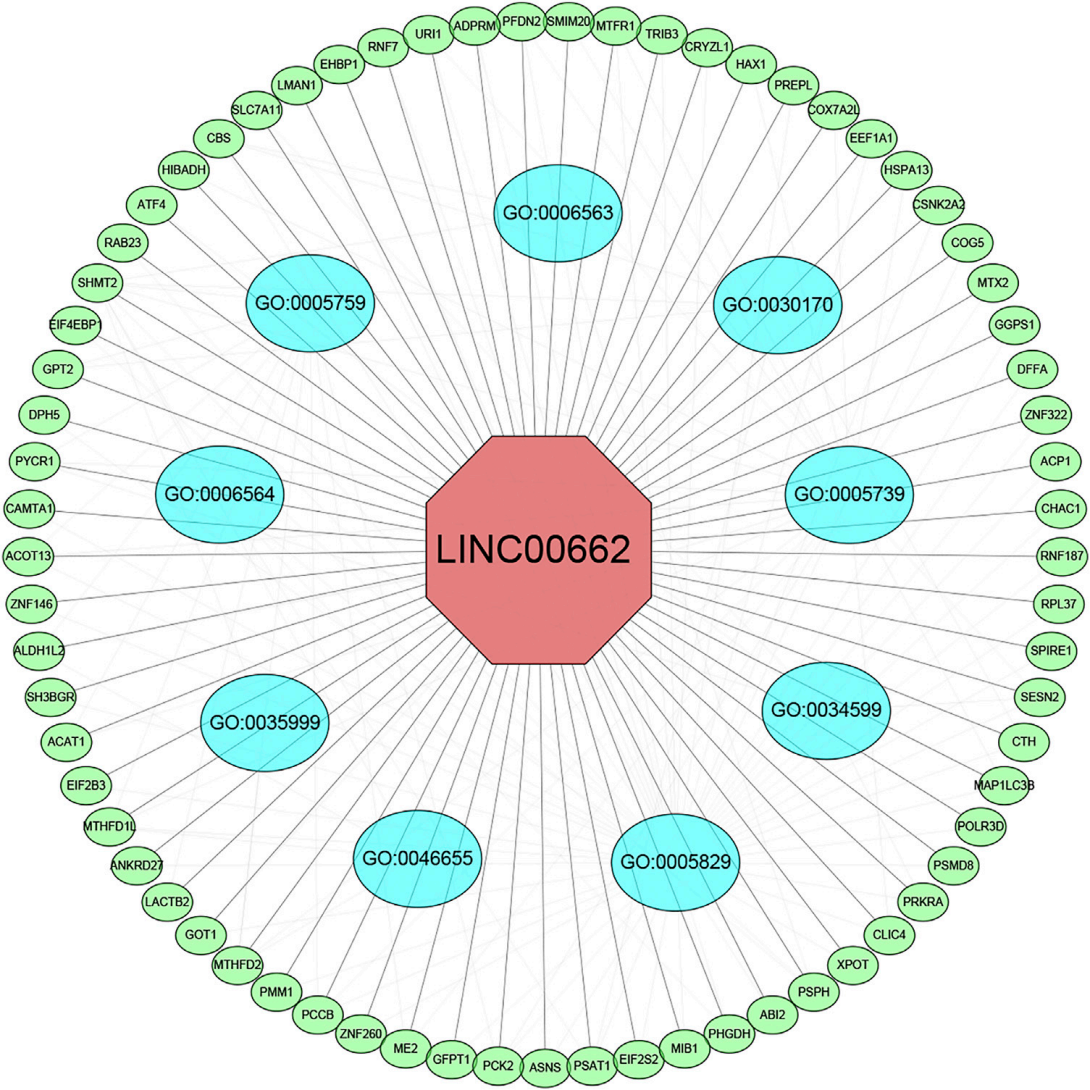
LINC00662-related genes interaction network analysis. Green nodes represent target genes and sky blue nodes represent the related pathway. As indicated in red, LINC00662 localized at the center of the network. GO:0005759 (mitochondrial matrix); GO:0006563 (L-serine metabolic process); GO:0030170 (pyridoxal phosphate binding); GO:0005739 (mitochondrion); GO:0034599 (cellular response to oxidative stress); GO:0005829 (cytosol); GO:0046655 (folic acid metabolic process); GO:0035999 (tetrahydrofolate interconversion); GO:0006564 (L-serine biosynthetic process).

We screened 200 LINC00662-related genes from the MEM database for GO and KEGG pathway analyses (Figure 7; Table 4). The GO analysis revealed that these genes were remarkably correlated with the cellular amino acid biosynthetic process, mitochondrial matrix, and transaminase activity. Additionally, the KEGG pathway analysis demonstrated that these genes were strongly interrelated with amino acid metabolic pathways. Furthermore, we built a signaling pathway network with Cytoscape (Figure 8).

**TABLE 4:** Gene ontology analysis of LINC00662-related genes.

GO number	Description	Genes	*p*-Value
GO:0005759	mitochondrial matrix	HIBADH, SHMT2, GPT2, PYCR1, ACOT13, ALDH1L2, ACAT1, MTHFD1L, LACTB2, MTHFD2, PCCB, ME2, PCK2	3.44716E-06
GO:0006564	L-serine biosynthetic process	PSAT1, SHMT2, PHGDH, PSPH	1.1503E-05
GO:0005829	Cytosol	CLIC4, PSMD8, MAP1LC3B, SESN2, PHGDH, RPL37, CHAC1, ACP1, ZNF322, PSPH, DFFA, GGPS1, COG5, CSNK2A2, ACOT13, EEF1A1, PREPL, CRYZL1, PCCB, TRIB3, MTFR1, PFDN2, ADPRM, URI1, SHMT2, RNF7, EHBP1, LMAN1, MTHFD1L, CBS, RAB23, EIF4EBP1, DPH5, CAMTA1, ZNF146, SH3BGR, PCK2, EIF2B3, ANKRD27, GOT1, PMM1, ZNF260, GFPT1, ASNS, EIF2S2, MIB1, ABI2, XPOT, PSAT1, PRKRA, POLR3D, CTH, SPIRE1, RNF187	2.99414E-05
GO:0030170	pyridoxal phosphate binding	GOT1, PSAT1, SHMT2, CBS, CTH, GPT2	3.0397E-05
GO:0005739	Mitochondrion	CLIC4, URI1, HIBADH, SHMT2, MTX2, PYCR1, ACOT13, ALDH1L2, HSPA13, ACAT1, COX7A2L, MAP1LC3B, HAX1, MTHFD1L, MTHFD2, PCCB, SESN2, PFDN2, MTFR1, ME2, SMIM20, PCK2	8.75681E-05
GO:0046655	folic acid metabolic process	MTHFD1L, SHMT2, MTHFD2, ALDH1L2	0.000222182
GO:0006563	L-serine metabolic process	SHMT2, CBS, PSPH	0.000526162
GO:0035999	tetrahydrofolate interconversion	MTHFD1L, SHMT2, MTHFD2	0.001553891
GO:0034599	cellular response to oxidative stress	PRKRA, SESN2, PYCR1, SLC7A11, ATF4	0.003681906

### Molecular mechanisms of LINC00662 carcinogenesis in various cancers

The molecular mechanisms of LINC00662-related carcinogenesis are recapitulated as follows.

• In HCC, LINC00662 facilitated the progression of HCC and the polarization of M2 macrophages by binding miR-107, miR-15a and miR-16 competitively to upregulate WNT3A expression and secretion, thereby activating the Wnt/β-catenin signaling pathway (Tian et al., 2020). Additionally, LINC00662 also exerted tumorigenic effects through simultaneous regulation of intracellular S-adenosylhomocysteine (SAH) and S-adenosylmethionine (SAM) levels resulting in altered methylation profiles (Guo et al., 2020).
• In lung cancer, LINC00662 affected lung cancer progression by modulating miR-320d/E2F1 and miR-145-5p/PAFAH1B2 axis (Lv et al., 2021; Xu et al., 2021).
• In CC, LINC00662 was involved in CC progression through regulation of miR-103a-3p/PDK4 and miR-497-5p/CDC25A (Wei et al., 2020; Liu Y. L. et al., 2021).
• In OSA, LINC00662 promoted OSA progression by sponging miR103a-3p and regulating SIK2 expression (Huang et al., 2021). In addition, LINC00662 could also affect OSA proliferation, invasion and migration by modulating miR-16-5p/ITPR1 and microRNA-15a-5p/Notch2 axis (Liu and Meng, 2020; Yu et al., 2021).
• In melanoma, LINC00662 promoted melanoma progression *via* sponging miR-890 to up-regulate ELK3 (Xia et al., 2020).
• In chordoma, LINC00662 facilitated chordoma progression *via* sponging miR-16-5p to activate RNF144B (Wang et al., 2020).
• In glioma, LINC00662 could be an oncogene by modulating the miR-107/HMGB1 and the miR-34a5p/LMAN2L axis (Geng et al., 2020; Wu et al., 2020).
• In CRC, LINC00662 facilitated colon cancer progression *via* binding miR-340-5p to upregulate CLDN8/IL22 expression and activating extracellular signal-regulated kinase (ERK) signaling pathway (Cheng B. et al., 2020). In addition, LINC00662 could also promote the development of CRC *via* modulating the miR497-5p/AVL9 axis (Wang et al., 2019).
• In OSCC, LINC00662 promoted the growth and metastasis of OSCC *via* miR-144-3p/EZH2 Axis (Liu P. et al., 2021). Besides, LINC00662 inflected the radiosensitivity of OSCC cells through hnRNPC-modulated AK4 (Chen Y. Z. et al., 2020).
• In OC, LINC00662 facilitated the survival and glycolysis of OC cells *via* miR-375 to modulate HIF-1α expression (Tao et al., 2020). In addition, LINC00662 promoted OC progression *via* activating the GRP78/P38 pathway (Wu et al., 2021).
• In PCa, LINC00662 promoted the metastasis and proliferation of PCa cells *via* modulating miR-34a (Li et al., 2019).
• In BC, LINC00662 facilitated the progression of BC cells through modulating the miR-497-5p/EglN2 axis (Cheng L. et al., 2020).
• In ESCC, LINC00662 promoted the progression of ESCC *via* sponging miR-3405p to activate HOXB2 (Zhang et al., 2020).
• In GC, LINC00662 accelerated GC progression *via* modulating the Hippo-YAP1 pathway (Liu et al., 2018). Additionally, LINC00662 facilitated the progression of GC progression *via* sponging miR-195-5p to up-regulate centrosomal protein 55 (Tao et al., 2022).
• In bladder cancer, facilitated bladder cancer progression *via* sponging miR-199a-5p (Ma et al., 2021).

## Discussion

The field of lncRNAs had received a lot of press attention lately because of the accelerated advancement of high-throughput sequencing technologies (Müller et al., 2015). Numerous studies have demonstrated that lncRNAs are implicated in tumorigenesis and development and are closely related to the prognosis of patients with tumors (Ge et al., 2018; Hu et al., 2021). In previous meta-analyses, FOXP4-AS1 (Zhang et al., 2022b), SNHG15 (Chen C. et al., 2020) and SNHG17 (Zhang et al., 2022a) were all linked to the clinicopathological characteristics and prognosis of various malignancies. These findings suggested that lncRNA could be a distinct cancer prognostic biomarker and therapeutic target.

LINC00662, a potential new cancer-related lncRNA, has been found to be upregulated in various malignancies and considered to function as a novel oncogene in tumor development. This anomalous expression typically resulted in poor clinical outcomes such as shorter OS and RFS, positive LNM, advanced tumor stage, and earlier DM (Li et al., 2019; Liu and Meng, 2020; Ma et al., 2021). In many malignancies, LINC00662 was also engaged in cell biological processes, including cell proliferation, migration, invasion, cell cycle arrest, and apoptosis inhibition (He et al., 2021). LINC00662 could act as a molecular sponge for miRNAs that influence tumor progression, and these miRNAs include miR-497-5p, miR-30b-3p, miR-103a-3p, etc. (Wang et al., 2019; Liu Y. L. et al., 2021; Wang et al., 2022). As a whole, these papers suggested that LINC00662 was linked to carcinogenesis and progression.

Because of the usefulness of LINC00662 in various cancers remains disputed, we conducted a meta-analysis to investigate the prognostic value and clinicopathological significance of LINC00662 differential expression in cancer patients. Our results suggested that LINC00662 overexpression was remarkably interrelated with worse OS in cancer patients and shorter RFS in patients with HCC. Moreover, further subgroup analysis indicated that LINC00662 overexpression was remarkably interrelated with worse OS in all subgroups, irrespective of the tumor type, follow-up time, HR availability and NOS score. Furthermore, patients with LINC00662 overexpression were more likely to have advanced tumor stage, larger tumor size, earlier LNM, and earlier DM. In a word, LINC00662 has an oncogenic role in tumors and may be a promising prognostic indicator. In addition, we used a public database to further confirm our results. LINC00662 overexpression was discovered in CHOL, DLBC, PCPG, SKCM, and THYM. Elevated LINC00662 expression was interrelated with worse OS in ACC, KICH, LGG, LIHC, SARC, THCA, and shorter DFS in ACC, KICH, LGG, LIHC. Collectively, the findings indicated that LINC00662 may be used as a novel biomarker for prognosis in cancer patients.

LINC00662 mainly acts through the Wnt/β-catenin signaling pathway, SMD signaling pathway, the ERK signaling pathway, and Hippo signaling pathway (Liu et al., 2018; Cheng B. et al., 2020; Tian et al., 2020). Furthermore, bioinformatics databases were used to forecast the functional mechanisms of LINC00662. GO and KEGG analyses indicated that the main biological functions of LINC00662-related genes were the cellular amino acid biosynthetic process, mitochondrial matrix, and transaminase activity, and that these genes were mainly significantly associated with amino acid metabolic pathways. These findings act as a reference for future studies on the mechanism of action of LINC00662.

Our meta-analysis has several limitations. Firstly, the sample size was relatively small and all incorporated items were conducted in China influenced the generalizability of the findings. To resolve this problem, we further verified our results by using public database to improve the credibility of our conclusions. Secondly, the HR values and 95% CIs for certain studies were obtained through KM curves extraction, which may have introduced some bias due to inaccurate calculation. Additionally, the cut-off of LINC00662 expression varied from study to study, which may have led to heterogeneity among studies.

## Conclusion

In summary, our study indicated that elevated LINC00662 expression is significantly correlated with shorter survival (including OS and RFS) and worse clinicopathological features (including tumor stage, tumor size, LNM, and DM). Therefore, LINC00662 could be a promising prognostic biomarker and therapeutic target in various cancers. Nevertheless, our conclusions require further confirmation through additional multicenter, high-quality, large-sample studies.

## Data Availability

The original contributions presented in the study are included in the article/supplementary material, further inquiries can be directed to the corresponding author.

## References

[B1] Avila M. A. Berasain C. Sangro B. Prieto J. (2006). New therapies for hepatocellular carcinoma. Oncogene 25 (27), 3866–3884. 10.1038/sj.onc.1209550 16799628

[B2] Chen C. Feng Y. Wang J. Liang Y. Zou W. (2020). Long non-coding RNA SNHG15 in various cancers: A meta and bioinformatic analysis. BMC Cancer 20 (1), 1156. 10.1186/s12885-020-07649-9 33243205 PMC7690101

[B3] Chen W. Li Y. Guo L. Zhang C. Tang S. (2021). Long non-coding RNA FTX predicts a poor prognosis of human cancers: A meta-analysis. Biosci. Rep. 41 (1), BSR20203995. 10.1042/bsr20203995 33398336 PMC7809557

[B4] Chen Y. Z. Bao C. C. Zhang X. X. Lin X. S. Fu Y. M. (2020). Knockdown of LINC00662 represses AK4 and attenuates radioresistance of oral squamous cell carcinoma. Cancer Cell Int. 20 (1), 244. 10.1186/s12935-020-01286-9 32549791 PMC7296632

[B5] Cheng B. Rong A. M. Zhou Q. B. Li W. L. (2020). LncRNA LINC00662 promotes colon cancer tumor growth and metastasis by competitively binding with miR-340-5p to regulate CLDN8/IL22 co-expression and activating ERK signaling pathway. J. Exp. Clin. Cancer Res. 39 (1), 5. 10.1186/s13046-019-1510-7 31900207 PMC6942292

[B6] Cheng L. Xing Z. H. Zhang P. Xu W. Q. (2020). Long non-coding RNA LINC00662 promotes proliferation and migration of breast cancer cells via regulating the miR-497-5p/EglN2 axis. Acta Biochim. Pol. 67 (2), 229–237. 10.18388/abp.2020_5203 32558530

[B7] Ge L. Tian J. H. Li Y. N. Pan J. X. Li G. Wei D. (2018). Association between prospective registration and overall reporting and methodological quality of systematic reviews: A meta-epidemiological study. J. Clin. Epidemiol. 93, 45–55. 10.1016/j.jclinepi.2017.10.012 29111471

[B8] Geng Y. B. Wu Y. L. Xu C. Li T. Zhang L. W. (2020). Long non-coding RNA LINC00662 regulated proliferation and migration by targeting miR-34a-5p/lman2l Axis in glioma. Onco. Targets. Ther. 13, 10161–10172. 10.2147/ott.S272616 33116598 PMC7553658

[B9] Gong W. J. Su Y. Liu Y. Sun P. Wang X. M. (2018). Long non-coding RNA Linc00662 promotes cell invasion and contributes to cancer stem cell-like phenotypes in lung cancer cells. J. Biochem. 164 (6), 461–469. 10.1093/jb/mvy078 30256974

[B10] Guo T. Gong C. Wu P. Battaglia-Hsu S. F. Feng J. Liu P. P. (2020). LINC00662 promotes hepatocellular carcinoma progression via altering genomic methylation profiles. Cell Death Differ. 27 (7), 2191–2205. 10.1038/s41418-020-0494-3 31959915 PMC7308394

[B11] He Y. Xu Y. Yu X. Sun Z. Guo W. (2021). The vital roles of LINC00662 in human cancers. Front. Cell Dev. Biol. 9, 711352. 10.3389/fcell.2021.711352 34354995 PMC8329443

[B12] Hu S. P. Ge M. X. Gao L. Jiang M. Hu K. W. (2021). LncRNA HCP5 as a potential therapeutic target and prognostic biomarker for various cancers: A meta-analysis and bioinformatics analysis. Cancer Cell Int. 21 (1), 686. 10.1186/s12935-021-02404-x 34923990 PMC8684676

[B13] Huang J. Lin F. Xu C. Xu Y. (2021). LINC00662 facilitates osteosarcoma progression via sponging miR-103a-3p and regulating SIK2 expression. J. Tissue Eng. Regen. Med. 15, 1082–1091. 10.1002/term.3242 34559955

[B14] Huarte M. Rinn J. L. (2010). Large non-coding RNAs: Missing links in cancer? Hum. Mol. Genet. 19 (R2), R152–R161. 10.1093/hmg/ddq353 20729297 PMC2953740

[B15] Li N. Zhang L. Y. Qiao Y. H. Song R. J. (2019). Long noncoding RNA LINC00662 functions as miRNA sponge to promote the prostate cancer tumorigenesis through targeting miR-34a. Eur. Rev. Med. Pharmacol. Sci. 23 (9), 3688–3698. 10.26355/eurrev_201905_17792 31114993

[B16] Lin Y. Shen Y. Chen J. Hu C. Zhou Z. Yuan C. (2021). The function of LncRNA FTX in several common cancers. Curr. Pharm. Des. 27 (20), 2381–2386. 10.2174/1381612826666201029164036 33121404

[B17] Liu P. P. Widjaja J. Dolo P. R. Yao L. Hong J. Shao Y. (2021). Comparing the anti-diabetic effect of sleeve gastrectomy with transit bipartition against sleeve gastrectomy and roux-en-Y gastric bypass using a diabetic rodent model. Obes. Surg. 31 (5), 2203–2210. 10.1007/s11695-021-05256-6 33507518

[B18] Liu S. H. Meng X. H. (2020). LINC00662 long non-coding RNA knockdown attenuates the proliferation, migration, and invasion of osteosarcoma cells by regulating the microRNA-15a-5p/notch2 Axis. Onco. Targets. Ther. 13, 7517–7530. 10.2147/ott.S256464 32848412 PMC7429411

[B19] Liu Y. L. Qiu S. Zheng X. L. Qiu Y. Y. Yao S. H. Ge Y. (2021). LINC00662 modulates cervical cancer cell proliferation, invasion, and apoptosis via sponging miR-103a-3p and upregulating PDK4. Mol. Carcinog. 60 (6), 365–376. 10.1002/mc.23294 33819358

[B20] Liu Z. Yao Y. Huang S. Li L. Jiang B. Guo H. (2018). LINC00662 promotes gastric cancer cell growth by modulating the Hippo-YAP1 pathway. Biochem. Biophys. Res. Commun. 505 (3), 843–849. 10.1016/j.bbrc.2018.09.191 30297104

[B21] Lv X. Lian Y. J. Liu Z. Y. Xiao J. G. Zhang D. H. Yin X. H. (2021). Exosomal long non-coding RNA LINC00662 promotes non-small cell lung cancer progression by miR-320d/E2F1 axis. Aging 13 (4), 6010–6024. 10.18632/aging.202522 33589572 PMC7950287

[B22] Ma X. Wen Y. Wang Y. Zhang M. Shi L. Wang C. (2021). Linc00662 plays an oncogenic role in bladder cancer by sponging miR-199a-5p. Am. J. Transl. Res. 13 (11), 12673–12683. 34956482 PMC8661171

[B23] Müller S. Raulefs S. Bruns P. Afonso-Grunz F. Plötner A. Thermann R. (2015). Next-generation sequencing reveals novel differentially regulated mRNAs, lncRNAs, miRNAs, sdRNAs and a piRNA in pancreatic cancer. Mol. Cancer 14, 94. 10.1186/s12943-015-0358-5 25910082 PMC4417536

[B24] Renganathan A. Felley-Bosco E. (2017). Long noncoding RNAs in cancer and therapeutic potential. Adv. Exp. Med. Biol. 1008, 199–222. 10.1007/978-981-10-5203-3_7 28815541

[B25] Sung H. Ferlay J. Siegel R. L. Laversanne M. Soerjomataram I. Jemal A. (2021). Global cancer statistics 2020: GLOBOCAN estimates of incidence and mortality worldwide for 36 cancers in 185 countries. Ca. Cancer J. Clin. 71 (3), 209–249. 10.3322/caac.21660 33538338

[B26] Tao F. Qi L. Liu G. (2022). Long intergenic non-protein coding RNA 662 accelerates the progression of gastric cancer through up-regulating centrosomal protein 55 by sponging microRNA-195-5p. Bioengineered 13 (2), 3007–3018. 10.1080/21655979.2021.2023978 35037833 PMC8974125

[B27] Tao L. M. Gong Y. F. Yang H. M. Pei J. H. Zhao X. J. Liu S. S. (2020). LINC00662 promotes glycolysis and cell survival by regulating miR- 375/HIF-1α axis in ovarian cancer. J. Biol. Regul. Homeost. Agents 34 (3), 467–477. 10.23812/19-300-A-18 32476381

[B28] Tian X. Wu Y. Yang Y. Wang J. Niu M. Gao S. (2020). Long noncoding RNA LINC00662 promotes M2 macrophage polarization and hepatocellular carcinoma progression via activating Wnt/β-catenin signaling. Mol. Oncol. 14 (2), 462–483. 10.1002/1878-0261.12606 31785055 PMC6998656

[B29] Wang B. Xu Z. Wang X. Xia S. Cai P. Wang M. (2022). Knockdown of lncRNA LINC00662 suppresses malignant behaviour of osteosarcoma cells via competition with miR-30b-3p to regulate ELK1 expression. J. Orthop. Surg. Res. 17 (1), 74. 10.1186/s13018-022-02964-2 35123530 PMC8818160

[B30] Wang C. B. Wang Y. Wang J. J. Guo X. L. (2020). LINC00662 triggers malignant progression of chordoma by the activation of RNF144B via targeting miR-16-5p. Eur. Rev. Med. Pharmacol. Sci. 24 (3), 1007–1022. 10.26355/eurrev_202002_20151 32096180

[B31] Wang H. Yu M. Hu W. Chen X. Luo Y. Lin X. (2019). Linc00662 promotes tumorigenesis and progression by regulating miR-497-5p/AVL9 Axis in colorectal cancer. Front. Genet. 10, 1385. 10.3389/fgene.2019.01385 32038723 PMC6993758

[B32] Wang Y. Fu L. Lu T. Zhang G. Zhang J. Zhao Y. (2021). Clinicopathological and prognostic significance of long non-coding RNA miat in human cancers: A review and meta-analysis. Front. Genet. 12, 729768. 10.3389/fgene.2021.729768 34659354 PMC8514773

[B33] Wei J. M. Wang L. L. Sun Y. L. Bao Y. X. (2020). LINC00662contributes to the progression and the radioresistance of cervical cancer by regulatingmiR-497-5p andCDC25A. Cell biochem. Funct. 38 (8), 1139–1151. 10.1002/cbf.3580 32869878

[B34] Wu J. S. Guo X. L. Xu D. X. Zhang H. R. (2020). LINC00662 sponges miR-107 accelerating the invasiveness and proliferation of glioma cells. J. Cancer 11 (19), 5700–5712. 10.7150/jca.46381 32913464 PMC7477458

[B35] Wu Y. Guo Q. H. Ju X. Z. Hu Z. X. Xia L. F. Deng Y. (2021). HNRNPH1-stabilized LINC00662 promotes ovarian cancer progression by activating the GRP78/p38 pathway. Oncogene 40 (29), 4770–4782. 10.1038/s41388-021-01884-5 34148056 PMC8298204

[B36] Xia X. Q. Lu W. L. Ye Y. Y. Chen J. (2020). LINC00662 promotes cell proliferation, migration and invasion of melanoma by sponging miR-890 to upregulate ELK3. Eur. Rev. Med. Pharmacol. Sci. 24 (16), 8429–8438. 10.26355/eurrev_202008_22640 32894549

[B37] Xu Z. Y. Peng J. Shi Z. Z. Chen X. L. Cheng H. Z. Wang H. (2021). Silencing linc00662 inhibits cell proliferation and colony formation of lung cancer cells via regulating the miR-145-5p-PAFAH1B2 axis. Biochem. Cell Biol. 99 (3), 330–338. 10.1139/bcb-2019-0396 33108738

[B38] Xue M. Zhuo Y. Shan B. (2017). MicroRNAs, long noncoding RNAs, and their functions in human disease. Methods Mol. Biol. 1617, 1–25. 10.1007/978-1-4939-7046-9_1 28540673

[B39] Yu M. Lu W. Cao Z. Xuan T. (2021). LncRNA LINC00662 exerts an oncogenic effect on osteosarcoma by the miR-16-5p/ITPR1 Axis. J. Oncol. 2021, 8493431. 10.1155/2021/8493431 34621314 PMC8492273

[B40] Zhang G. Fu L. Wang Y. Liu B. Ma S. Ma H. (2022a). Integrative pan-cancer analysis indicates the prognostic importance of long noncoding RNA SNHG17 in human cancers. Pathol. Res. Pract. 238, 154140. 10.1016/j.prp.2022.154140 36167008

[B41] Zhang G. Wang Y. Han X. Lu T. Fu L. Jin H. (2022b). FOXP4-AS1 may be a potential prognostic biomarker in human cancers: A meta-analysis and bioinformatics analysis. Front. Oncol. 12, 799265. 10.3389/fonc.2022.799265 35719909 PMC9204280

[B42] Zhang Z. M. Liang X. Y. Ren L. Zhang S. X. Li S. Y. Wan T. X. (2020). LINC00662promotes cell viability and metastasis in esophageal squamous cell carcinoma by spongingmiR-340-5p and upregulatingHOXB2. Thorac. Cancer 11 (8), 2306–2315. 10.1111/1759-7714.13551 32633082 PMC7396358

[B43] Zhong C. Zhang Q. Zhang M. Qi Y. Duan S. (2021). LINC00662: A new oncogenic lncRNA with great potential. J. Cell. Physiol. 237, 1105–1118. 10.1002/jcp.30599 34647332

